# 2-(2,4-Di­nitro­phen­yl)-1-(pyridin-4-yl)ethanol monohydrate

**DOI:** 10.1107/S2414314620016405

**Published:** 2021-02-02

**Authors:** Houshi Huang, Zhichao Wu

**Affiliations:** a Department of Chemistry, Anhui University, Hefei, Anhui 230039, People’s Republic of China; University of Aberdeen, Scotland

**Keywords:** crystal structure, dihedral angle, hydrogen bonds, layered structure

## Abstract

In the title compound, C_13_H_11_N_3_O_5_·H_2_O, the dihedral angle between the aromatic rings is 9.60 (7)° and the chain linking the rings has an *anti* conformation with a torsion angle of −178.28 (12)°. In the crystal, the components are linked by O—H⋯O and O—H⋯N hydrogen bonds, generating (010) sheets.

## Abstract 2

In the title compound, the dihedral angle between the aromatic rings is 9.60 (7)° and the chain linking the rings has an *anti* conformation with a torsion angle of −178.28 (12)°. In the crystal, the components are linked by O—H⋯O and O—H⋯N hydrogen bonds, generating (010) sheets.

## Graphical abstract

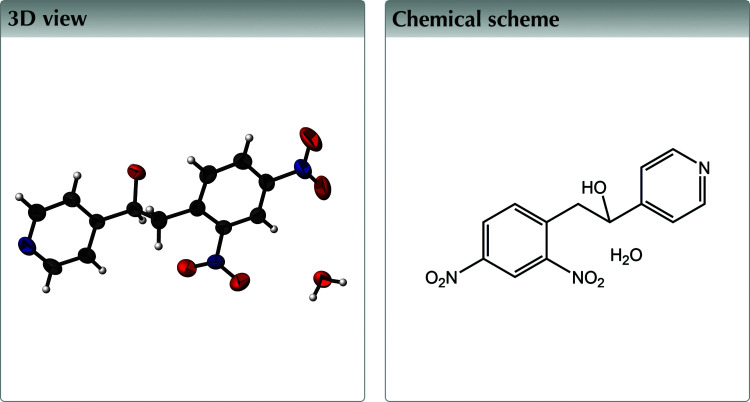

## Structure description

**Figure 1:**
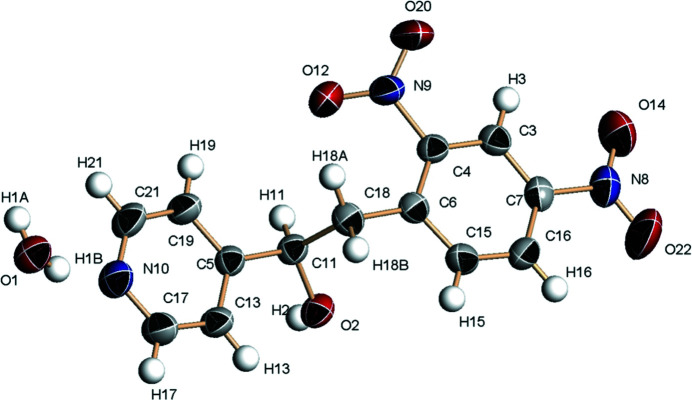
The mol­ecular structure of the title compound showing 50% displacement ellipsoids.

Pyridine derivatives have been widely used in biochemistry. Pyridine salts, for example, are well known for their photoactivity and exhibit potential for targetting mitochondria and in the photodynamic therapy of diseases (Wang *et al.*, 2020 ▸; Li *et al.*,2017 ▸). The Knoevenagel reaction is one of the most efficient methods of constructing pyridine-containing organic semiconductors though a two-step process: (1) format the hydroxyl-containing inter­mediates; (2) obtain products by a dehydration reaction. We report here the of the title compound, C_13_H_11_N_3_O_5_·H_2_O (Fig. 1 ▸), a hydroxyl-containing inter­mediate. The dihedral angle between the benzene and pyridine rings is 9.60 (7)° and the chain linking the rings has an *anti* conformation with a C5—C11—C18—C6 torsion angle of −178.28 (12)°. In the crystal, the components are linked by O—H⋯O and O—H⋯N hydrogen bonds (Table 1 ▸), generating (010) sheets.

## Synthesis and crystallization

1-Methyl-2,4-di­nitro-benzene (0.546 g, 3.00 mmol) and pyridine-4-carbaldehyde (0.321 g, 3.00 mmol) were dissolved in dimethyl sulfoxide (50 ml). The mixture was heated to 80°C for 4 h, then cooled to room temperature and poured into water. The precipitate was collected by filtration and dried to obtain a yellow solid (0.712 g, 2.4 mol). Yellow crystals suitable for X-ray analysis were obtained by recrystallization from ethanol solution.

## Refinement

Crystal data, data collection and structure refinement details are summarized in Table 2 ▸.

## Supplementary Material

Crystal structure: contains datablock(s) I. DOI: 10.1107/S2414314620016405/hb4371sup1.cif

Structure factors: contains datablock(s) I. DOI: 10.1107/S2414314620016405/hb4371Isup2.hkl

Supporting information file. DOI: 10.1107/S2414314620016405/hb4371Isup3.cml

CCDC reference: 2047186

Additional supporting information:  crystallographic information; 3D view; checkCIF report

## Figures and Tables

**Table 1:** Hydrogen-bond geometry (Å, °)

*D*—H⋯*A*	*D*—H	H⋯*A*	*D*⋯*A*	*D*—H⋯*A*
O1—H1*A*⋯O2^i^	0.80 (2)	2.01 (2)	2.801 (3)	173 (2)
O1—H1*B*⋯N10^ii^	0.86 (3)	2.00 (3)	2.841 (3)	166.1 (19)
O2—H2⋯O1^iii^	0.82	1.85	2.666 (3)	175
C17—H17⋯O20^iv^	0.93	2.53	3.226 (4)	131

Symmetry codes: (i) 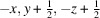 ; (ii) 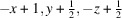 ; (iii) 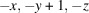 ; (iv) 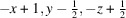 .

**Table 2:** Experimental details

Crystal data	
Chemical formula	C_13_H_11_N_3_O_5_·H_2_O
*M*_r_	307.26
Crystal system, space group	Monoclinic, *P*2_1_/*c*
Temperature (K)	296
*a*, *b*, *c* (Å)	8.639 (8), 20.045 (12), 8.116 (5)
β (°)	101.326 (5)
*V* (Å^3^)	1378.2 (17)
*Z*	4
Radiation type	Mo *K*α
μ (mm^−1^)	0.12
Crystal size (mm)	0.19 × 0.18 × 0.17

Data collection	
Diffractometer	Bruker SMART CCD
Absorption correction	Multi-scan (*SADABS*; Bruker, 2004 ▸)
*T*_min_, *T*_max_	0.616, 0.746
No. of measured, independent and observed [*I* > 2σ(*I*)] reflections	10488, 2889, 2531
*R*_int_	0.025
(sin θ/λ)_max_ (Å^−1^)	0.645

Refinement	
*R*[*F* ^2^ > 2σ(*F* ^2^)], *wR*(*F* ^2^), *S*	0.045, 0.125, 1.03
No. of reflections	2889
No. of parameters	208
H-atom treatment	H atoms treated by a mixture of independent and constrained refinement
Δρ_max_, Δρ_min_ (e Å^−3^)	0.37, −0.24

Computer programs: *SMART* and *SAINT* (Bruker, 2004 ▸), *SHELXS97* (Sheldrick, 2008 ▸), *SHELXL2014/7* (Sheldrick, 2015 ▸) and *SHELXTL* (Sheldrick, 2008 ▸).

## full crystallographic data

### Crystal data

**Table:**

C_13_H_11_N_3_O_5_·H_2_O	*F*(000) = 640
*M**_r_* = 307.26	*D*_x_ = 1.481 Mg m^−^^3^
Monoclinic, *P*2_1_/*c*	Mo *K*α radiation, λ = 0.71073 Å
*a* = 8.639 (8) Å	Cell parameters from 6924 reflections
*b* = 20.045 (12) Å	θ = 2.6–27.1°
*c* = 8.116 (5) Å	µ = 0.12 mm^−^^1^
β = 101.326 (5)°	*T* = 296 K
*V* = 1378.2 (17) Å^3^	Block, yellow
*Z* = 4	0.19 × 0.18 × 0.17 mm

### Data collection

**Table:**

Bruker SMART CCD diffractometer	2531 reflections with *I* > 2σ(*I*)
Radiation source: sealed tube	*R*_int_ = 0.025
ω scans	θ_max_ = 27.3°, θ_min_ = 2.0°
Absorption correction: multi-scan (SADABS; Bruker, 2004)	*h* = −11→11
*T*_min_ = 0.616, *T*_max_ = 0.746	*k* = −22→25
10488 measured reflections	*l* = −10→10
2889 independent reflections	

### Refinement

**Table:**

Refinement on *F*^2^	Primary atom site location: structure-invariant direct methods
Least-squares matrix: full	Hydrogen site location: mixed
*R*[*F*^2^ > 2σ(*F*^2^)] = 0.045	H atoms treated by a mixture of independent and constrained refinement
*wR*(*F*^2^) = 0.125	*w* = 1/[σ^2^(*F*_o_^2^) + (0.0605*P*)^2^ + 0.4306*P*] where *P* = (*F*_o_^2^ + 2*F*_c_^2^)/3
*S* = 1.03	(Δ/σ)_max_ = 0.001
2889 reflections	Δρ_max_ = 0.37 e Å^−^^3^
208 parameters	Δρ_min_ = −0.24 e Å^−^^3^
0 restraints	

### Special details

**Table:**

Geometry. All esds (except the esd in the dihedral angle between two l.s. planes) are estimated using the full covariance matrix. The cell esds are taken into account individually in the estimation of esds in distances, angles and torsion angles; correlations between esds in cell parameters are only used when they are defined by crystal symmetry. An approximate (isotropic) treatment of cell esds is used for estimating esds involving l.s. planes.

### Fractional atomic coordinates and isotropic or equivalent isotropic displacement parameters (Å^2^)

**Table:**

	*x*	*y*	*z*	*U*_iso_*/*U*_eq_	
O2	0.11854 (12)	0.30340 (6)	0.04733 (14)	0.0475 (3)	
H2	0.0856	0.3009	−0.0544	0.071*	
C3	−0.03252 (17)	0.51894 (7)	0.25550 (18)	0.0418 (3)	
H3	−0.0432	0.5644	0.2332	0.050*	
C4	0.10032 (16)	0.48431 (7)	0.23286 (18)	0.0389 (3)	
C5	0.40549 (16)	0.30336 (7)	0.08026 (17)	0.0383 (3)	
C6	0.12367 (17)	0.41637 (7)	0.26901 (17)	0.0393 (3)	
C7	−0.14831 (17)	0.48357 (8)	0.31235 (18)	0.0435 (3)	
N8	−0.29271 (16)	0.51917 (8)	0.33273 (18)	0.0563 (4)	
N9	0.21854 (15)	0.52431 (6)	0.16714 (18)	0.0500 (3)	
N10	0.68428 (15)	0.23063 (7)	0.09129 (19)	0.0534 (4)	
C11	0.25687 (16)	0.34380 (7)	0.07980 (18)	0.0394 (3)	
H11	0.2482	0.3787	−0.0060	0.047*	
O12	0.30318 (16)	0.49757 (7)	0.08429 (19)	0.0669 (4)	
C13	0.41333 (17)	0.23595 (7)	0.1180 (2)	0.0451 (3)	
H13	0.3254	0.2137	0.1408	0.054*	
O14	−0.30398 (18)	0.57810 (8)	0.2980 (2)	0.0795 (4)	
C15	0.0034 (2)	0.38446 (8)	0.3309 (2)	0.0488 (4)	
H15	0.0154	0.3396	0.3594	0.059*	
C16	−0.1328 (2)	0.41660 (8)	0.3516 (2)	0.0504 (4)	
H16	−0.2119	0.3937	0.3910	0.060*	
C17	0.55312 (19)	0.20209 (8)	0.1213 (2)	0.0529 (4)	
H17	0.5560	0.1567	0.1459	0.064*	
C18	0.26799 (18)	0.37632 (7)	0.25304 (19)	0.0438 (3)	
H18A	0.2839	0.3417	0.3382	0.053*	
H18B	0.3596	0.4054	0.2747	0.053*	
C19	0.54071 (18)	0.33341 (8)	0.0472 (2)	0.0499 (4)	
H19	0.5407	0.3785	0.0205	0.060*	
O20	0.2244 (2)	0.58322 (7)	0.1986 (3)	0.1047 (7)	
C21	0.67505 (19)	0.29564 (9)	0.0543 (2)	0.0547 (4)	
H21	0.7647	0.3166	0.0320	0.066*	
O22	−0.3925 (2)	0.48738 (9)	0.3855 (3)	0.1002 (6)	
O1	−0.00579 (15)	0.69790 (7)	0.28295 (16)	0.0537 (3)	
H1A	−0.044 (3)	0.7282 (11)	0.324 (3)	0.072 (7)*	
H1B	0.094 (3)	0.7007 (10)	0.319 (3)	0.072 (6)*	

### Atomic displacement parameters (Å^2^)

**Table:**

	*U*^11^	*U*^22^	*U*^33^	*U*^12^	*U*^13^	*U*^23^
O2	0.0343 (5)	0.0568 (6)	0.0508 (6)	−0.0054 (4)	0.0066 (4)	−0.0012 (5)
C3	0.0400 (7)	0.0391 (7)	0.0445 (7)	0.0017 (6)	0.0040 (6)	−0.0014 (6)
C4	0.0356 (7)	0.0387 (7)	0.0413 (7)	−0.0043 (5)	0.0053 (5)	−0.0016 (6)
C5	0.0340 (7)	0.0411 (7)	0.0395 (7)	−0.0008 (5)	0.0064 (5)	−0.0012 (5)
C6	0.0403 (7)	0.0395 (7)	0.0377 (7)	0.0010 (6)	0.0062 (5)	−0.0019 (5)
C7	0.0380 (7)	0.0513 (8)	0.0412 (7)	0.0024 (6)	0.0075 (6)	−0.0072 (6)
N8	0.0428 (7)	0.0717 (10)	0.0559 (8)	0.0063 (7)	0.0134 (6)	−0.0074 (7)
N9	0.0424 (7)	0.0421 (7)	0.0661 (9)	−0.0068 (5)	0.0121 (6)	−0.0001 (6)
N10	0.0366 (7)	0.0576 (8)	0.0659 (9)	0.0065 (6)	0.0094 (6)	0.0050 (7)
C11	0.0337 (7)	0.0408 (7)	0.0433 (7)	0.0001 (5)	0.0065 (5)	0.0032 (6)
O12	0.0645 (8)	0.0615 (8)	0.0841 (9)	−0.0096 (6)	0.0378 (7)	−0.0020 (7)
C13	0.0352 (7)	0.0427 (8)	0.0577 (9)	−0.0029 (6)	0.0095 (6)	0.0028 (6)
O14	0.0669 (9)	0.0698 (9)	0.1059 (12)	0.0257 (7)	0.0271 (8)	0.0022 (8)
C15	0.0599 (9)	0.0382 (7)	0.0521 (8)	−0.0003 (7)	0.0200 (7)	0.0022 (6)
C16	0.0502 (9)	0.0520 (9)	0.0534 (9)	−0.0089 (7)	0.0212 (7)	−0.0031 (7)
C17	0.0424 (8)	0.0441 (8)	0.0718 (11)	0.0034 (6)	0.0100 (7)	0.0057 (7)
C18	0.0423 (8)	0.0433 (8)	0.0442 (8)	0.0061 (6)	0.0049 (6)	0.0004 (6)
C19	0.0407 (8)	0.0444 (8)	0.0661 (10)	−0.0025 (6)	0.0144 (7)	0.0063 (7)
O20	0.0977 (12)	0.0435 (7)	0.193 (2)	−0.0191 (7)	0.0773 (13)	−0.0134 (10)
C21	0.0345 (8)	0.0607 (10)	0.0708 (11)	−0.0036 (7)	0.0149 (7)	0.0058 (8)
O22	0.0645 (9)	0.1094 (13)	0.1428 (17)	0.0092 (9)	0.0599 (11)	0.0136 (12)
O1	0.0379 (6)	0.0607 (8)	0.0601 (7)	−0.0024 (5)	0.0034 (5)	−0.0086 (6)

### Geometric parameters (Å, º)

**Table:**

O2—C11	1.4248 (19)	N10—C17	1.334 (2)
O2—H2	0.8200	N10—C21	1.336 (2)
C3—C7	1.378 (2)	C11—C18	1.536 (2)
C3—C4	1.384 (2)	C11—H11	0.9800
C3—H3	0.9300	C13—C17	1.381 (2)
C4—C6	1.400 (2)	C13—H13	0.9300
C4—N9	1.478 (2)	C15—C16	1.380 (2)
C5—C13	1.384 (2)	C15—H15	0.9300
C5—C19	1.387 (2)	C16—H16	0.9300
C5—C11	1.518 (2)	C17—H17	0.9300
C6—C15	1.395 (2)	C18—H18A	0.9700
C6—C18	1.510 (2)	C18—H18B	0.9700
C7—C16	1.380 (2)	C19—C21	1.377 (2)
C7—N8	1.474 (2)	C19—H19	0.9300
N8—O14	1.214 (2)	C21—H21	0.9300
N8—O22	1.216 (2)	O1—H1A	0.80 (2)
N9—O20	1.207 (2)	O1—H1B	0.86 (3)
N9—O12	1.2113 (19)		
			
C11—O2—H2	109.5	C5—C11—H11	109.3
C7—C3—C4	117.52 (14)	C18—C11—H11	109.3
C7—C3—H3	121.2	C17—C13—C5	119.26 (14)
C4—C3—H3	121.2	C17—C13—H13	120.4
C3—C4—C6	123.32 (13)	C5—C13—H13	120.4
C3—C4—N9	115.20 (13)	C16—C15—C6	122.89 (15)
C6—C4—N9	121.48 (13)	C16—C15—H15	118.6
C13—C5—C19	117.41 (14)	C6—C15—H15	118.6
C13—C5—C11	121.77 (13)	C15—C16—C7	118.20 (14)
C19—C5—C11	120.79 (14)	C15—C16—H16	120.9
C15—C6—C4	115.76 (13)	C7—C16—H16	120.9
C15—C6—C18	118.25 (14)	N10—C17—C13	123.82 (16)
C4—C6—C18	125.94 (13)	N10—C17—H17	118.1
C3—C7—C16	122.26 (14)	C13—C17—H17	118.1
C3—C7—N8	118.34 (15)	C6—C18—C11	113.58 (12)
C16—C7—N8	119.40 (14)	C6—C18—H18A	108.8
O14—N8—O22	124.18 (16)	C11—C18—H18A	108.8
O14—N8—C7	118.42 (15)	C6—C18—H18B	108.8
O22—N8—C7	117.39 (17)	C11—C18—H18B	108.8
O20—N9—O12	123.07 (15)	H18A—C18—H18B	107.7
O20—N9—C4	117.22 (14)	C21—C19—C5	119.22 (15)
O12—N9—C4	119.71 (13)	C21—C19—H19	120.4
C17—N10—C21	116.36 (14)	C5—C19—H19	120.4
O2—C11—C5	112.04 (13)	N10—C21—C19	123.92 (15)
O2—C11—C18	108.06 (12)	N10—C21—H21	118.0
C5—C11—C18	108.88 (11)	C19—C21—H21	118.0
O2—C11—H11	109.3	H1A—O1—H1B	106 (2)

### Hydrogen-bond geometry (Å, º)

**Table:**

*D*—H···*A*	*D*—H	H···*A*	*D*···*A*	*D*—H···*A*
O1—H1*A*···O2^i^	0.80 (2)	2.01 (2)	2.801 (3)	173 (2)
O1—H1*B*···N10^ii^	0.86 (3)	2.00 (3)	2.841 (3)	166.1 (19)
O2—H2···O1^iii^	0.82	1.85	2.666 (3)	175
C17—H17···O20^iv^	0.93	2.53	3.226 (4)	131

Symmetry codes: (i) −*x*, *y*+1/2, −*z*+1/2; (ii) −*x*+1, *y*+1/2, −*z*+1/2; (iii) −*x*, −*y*+1, −*z*; (iv) −*x*+1, *y*−1/2, −*z*+1/2.
